# Liposome-Encapsulated Baicalein Suppressed Lipogenesis and Extracellular Matrix Formation in Hs68 Human Dermal Fibroblasts

**DOI:** 10.3389/fphar.2018.00155

**Published:** 2018-03-06

**Authors:** Chien-Liang Fang, Yiwei Wang, Kevin H.-Y. Tsai, Hsin-I Chang

**Affiliations:** ^1^Division of Plastic and Reconstructive Surgery, Department of Surgery, Ditmanson Medical Foundation Chia-Yi Christian Hospital, Chiayi City, Taiwan; ^2^Burns Research Group, ANZAC Research Institute, Concord Hospital, University of Sydney, Concord, NSW, Australia; ^3^Department of Biochemical Science and Technology, National Chiayi University, Chiayi City, Taiwan

**Keywords:** baicalein, liposomes, Hs68 human dermal fibroblast, adipogenic differentiation medium, lipogenesis

## Abstract

The dermis of human skin contains large numbers of fibroblasts that are responsible for the production of the extracellular matrix (ECM) that supporting skin integrity, elasticity and wound healing. Previously, an *in vivo* study demonstrated that dermal fibroblasts siting in the lower dermis are capable to convert into skin adipose layer and hence fibroblast lipogenesis may vary the structure and elasticity of dermis. In the present study, Hs68 human dermal fibroblasts were utilized as an *in vitro* model to study the lipogenesis via using adipogenic differentiation medium (ADM). Baicalein, isolated from *Scutellaria baicalensis*, is one of the flavonoids to inhibit adipocyte differentiation due to high antioxidant activity *in vitro*. In order to develop a suitable formulation for baicalein (a poorly water-soluble drug), soybean phosphatidylcholine (SPC) was used to prepare baicalein-loaded liposomes to enhance drug bioavailability. Our results demonstrated that liposome-encapsulated baicalein protected cell viability and increased cellular uptake efficiency of Hs68 fibroblasts. Lipid accumulation, triglyceride synthesis and gene expressions of lipogenesis enzymes (FABP4 and LPL) were significantly increased in ADM-stimulated Hs68 fibroblasts but subsequently suppressed by liposome-encapsulated baicalein. In addition, ADM-induced TNF-α expression and related inflammatory factors was down-regulated by liposome-encapsulated baicalein. Through ADM-induced lipogenesis, the protein expression of elastin, type I and type III collagens increased remarkably, whereas liposome-encapsulated baicalein can down-regulate ADM-induced ECM protein synthesis. Taken together, we found that liposome-encapsulated baicalein can inhibit ADM-induced lipid accumulation and ECM formation in Hs68 fibroblasts through the suppression of lipogenesis enzymes and inflammatory responses. Liposome-encapsulated baicalein may have the potential to improve wound healing and restore skin structure after skin injury.

## Introduction

Dermis in skin is the layer lies between epidermis and subcutaneous adipose tissue. Dermis is composed of fibrous and elastic tissue, thereby providing strength and flexibility to skin. Dermis is the thickest layer of the skin, but that can be varied based on body mass index (BMI), gender and locations. Recently, many researches revealed the relationship between adipose tissue in correlation with dermal fibroblasts. Ezure and Amano (2010) and Hew et al. (2016) demonstrated that the subcutaneous adipose layer of mice was remarkably thickened after being fed with high-fat diet, while the dermal layer was thinned (Ezure and Amano, 2010; Hew et al., 2016). Hence, such increase of adipose tissue may reduce the proliferation of dermal fibroblasts and elasticity of skin (Ezure and Amano, 2010). Moreover, adipogenesis-defect animal model indicated that intradermal adipocytes could mediate fibroblast recruitment during skin wound healing (Schmidt and Horsley, 2013). Interestingly, Wojciechowicz et al. (2013) confirmed that dermal fibroblasts siting in the lower dermis are capable to convert into skin adipose layer independently without influence from subcutaneous adipose tissue. Furthermore, the thickness of the lower dermis increased was found concomitant with the extension and downgrowth of hair follicles. Therefore, a key objective of studying the lipogenesis in Hs68 fibroblasts is to determine if and how lipogenesis can modulate inflammatory responses and change extracellular matrix (ECM) structure and composition in human dermal fibroblasts.

Dermal fibroblasts are the major cell population in dermis which are responsible for ECM production and wound healing. In the study reported by Rakar et al. (2012) primary human dermal fibroblasts were found to differentiate toward adipocytes, osteoblasts and chondrocytes using different induction media. Hence, relatively undifferentiated fibroblasts can express a particular phenotype depend on physiological stimuli and microenvironmental factors to which they are exposed (Koumas et al., 2003). This discovery supports the concept of fibroblast plasticity and proposes that fibroblasts can be transformed into adipocytes. In previous report suing human neonatal and adult lung tissues, alveolar interstitial fibroblasts were detected with the presence of lipid and the expression of adipocyte differentiation-related protein (ADRP), a protein necessary for lipid uptake, leading to their classification as lipid-containing fibroblasts or lipofibroblasts (Rehan et al., 2006). Lung specimens harvesting from patients with idiopathic pulmonary fibrosis (IPF) were found to have decreased lipofibroblast marker expression compared with non-IPF control samples (Bhattacharya, 2016), resulting in a hypothesis that conversion of fibroblasts into lipid-containing cells or lipofibroblasts may be able to prevent tissue fibrosis, abnormal wound healing, and hypertrophic scars. However, in skin wound healing, the role of lipogenesis in dermal fibroblasts were under investigated and the mechanisms has yet to be defined. Our previous study confirmed that adipogenic differentiation medium (ADM) can stimulate the differentiation of Hs68 human dermal fibroblasts to adipocyte-like cells through the lipid accumulation and mRNA expressions of PPAR-γ, LPL and FABP4 (Fang et al., 2016). In the present study, we aim to further investigate the impact of lipogenesis on the inflammation responses and ECM formation in Hs68 fibroblasts.

Baicalein (5,6,7-trihydroxy-2-phenyl-4H-1-benzopyran-4-one), one of the remarkable flavonoids, is isolated from the root of *Scutellaria baicalensis*. Baicalein possesses a variety of biological activities, including high antioxidant, anti-inflammatory, anti-proliferative, anti-apoptotic and anti-tumor activities (Gao et al., 2013). Baicalein has been demonstrated to decrease skin thickness and to suppress the expression levels of matrix metalloproteinase (MMP)-9, and vascular endothelial growth factor (VEGF) in ultraviolet (UV) B irradiated skin of mice models (Kimura and Sumiyoshi, 2011). Moreover, baicalein is known to inhibit radiation induced expression of nuclear transcription factor nuclear factor kappa B (NF-κB) and Cyclooxygenase-2 (COX-2) in human keratinocytes (Kimura and Sumiyoshi, 2011). Seo et al. (2014) found that baicalein can inhibit lipid accumulation and adipocyte differentiation by suppressing adipogenic factors such as PPARγ and C/EBPα through m-TOR signaling pathway in 3T3-L1 fibroblasts. However, using baicalein in therapeutic application are limited due to its low water solubility and poor oral bioavailability (Huang et al., 2014; de Oliveira et al., 2015). Recently, Rajkumari et al. (2017) have used baicalein as a reducing and capping agent in the synthesis of gold nanoparticles to inhibit Pseudomonas aeruginosa PAO1 biofilm formation. Li X. et al. (2017) also synthesized amine-modified mesoporous silica nanoparticles to encapsulate baicalein to exhibit anti-inflammatory effect on primary human gingival epithelial cells. Interestingly, baicalein was found to reduce the cytotoxicity of ZnO nanoparticles in Caco-2 cells (Li Y. et al., 2017). Therefore, liposomal nanoencapsulation of baicalein was introduced and investigated aiming to improve therapeutic efficacy through the increase of drug solubility and cell absorption efficiency (Moulaoui et al., 2015)

Composite phospholipid liposomes, similar to the lipid bilayer of cell membrane, have been formulated in nano-scaled sizes to increase the *in vivo* bioavailability for hydrophobic drugs (Kalepu and Nekkanti, 2015; Ong et al., 2016). Liposomes can be modified with various lipids to enhance drug loading efficiency and release characteristics (Mohammed et al., 2004) with reduced cytotoxicity, better biocompatibility and stability (Zeng et al., 2016). Various techniques, such as Bangham method, the detergent depletion method, the ether/ethanol injection method, the reverse phase evaporation and the emulsion method are previously reported to formulate drug-loaded liposomes with high entrapment efficiency (EE), narrow polydiversity index (PDI) and long term stability (Galović Rengel et al., 2002; Bergstrand et al., 2003). Based on low aqueous solubility and high cytotoxicity of baicalein on Hs 68 human dermal fibroblasts, phosphatidylcholine (PC) -based liposomes were used to encapsulate baicalein. Thereafter, we investigated the inhibitory effects of liposome-encapsulated baicalein on the ADM-induced adipogenesis, inflammatory responses and ECM synthesis in human dermal fibroblasts, Hs68.

## Materials and Methods

### Chemicals and Cell Culture

Baicalein was purchased from Sigma-Aldrich, USA. Phospholipon 90G (phosphatidylcholine 90%) was acquired from American Lecithin Company, Germany. All cell culture materials including, Dulbecco’s modified eagle’s medium (DMEM), fetal bovine serum (FBS), L-glutamine, adipocyte differentiation medium (ADM) were obtained from Gibco (Grand Island, NY, United States). Oil red o staining kit was purchased from Lifeline Cell Technology (Frederick, MD, United States). Adipogenesis assay kit was purchased from Sigma–Aldrich (St. Louis, MO, United States). All reagents and solvents are for research use only. Human foreskin fibroblasts (Hs68) were obtained from ATCC (Manassas, VA, United States) and Murine macrophages RAW 264.7 were purchased from Bioresource collection and Research Center, Food Industry Research and Development Institute, Taiwan. Both cell lines were cultured in DMEM supplemented with 10% v/v FBS, 100 units/ml penicillin and 100 μg/ml of streptomycine under steady state condition at 37°C with 5% CO_2_ in a humidified incubator.

### Liposomal Formulation

Baicalein-loaded liposomes were generated using a thin-film hydration and size reduction procedure as previously described (Yeh et al., 2015). Briefly, 100 mg of phospholipids were dissolved in 8 ml of chloroform, series amounts (20–60 μg) of baicalein were dissolved in 2 ml of ethanol and mixed together in a round-bottom flask. The organic solvents were evaporated by using rotary evaporator (Eyela, N-1000, Japan) at 45°C prior to vacuum dry to form a dry lipid film. The lipid film was rehydrated by addition of 2 ml PBS (Phosphate buffered saline). Liposomes were then resized and uniformed through extruding polycarbonate membranes with series decreased pore sizes from 400 nm, 200 nm to 100 nm (Avanti Mini Extruderm, Alabaster, AL, United States). Empty liposomes were prepared by the same process with drug-free methanol.

### Particle Characterization

The particle stability of liposomes was identified by storing and measuring the particle sizes over 2 weeks. The particle sizes were measured by using a Dynamic Light Scattering Instrument (HORIBA, LB-550, Japan). The solution of baicalein-loaded liposomes was diluted in approximate 30 times with double-distilled water to make sure that the light scattering intensity was in the instrument’s detectable range. The Polydispersity index (PDI) and zeta potential of liposomes were determined by Dynamic Light Scattering Analyzer (Malvern, Malvern Nano-Zs, England).

### Entrapment Efficiency

Loading efficiency of baicalein in liposomes was assessed using a high-speed centrifugation once the liposomes were formulated. Baicalein-loaded liposomes were spun down at 80,000 rpm by Beckman ultra-high speed centrifuge for 30 min. Next, the supernatants were carefully discarded and pellets were subsequently redissolved in the equal volume of ethanol. The concentration of baicalein in liposomes were then measured using an ELISA reader (Tecan, infinite M200) at wavelength of 277 nm. The encapsulation efficiency of baicalein in liposomes was calculated from the standard curve. The entrapment efficiency (EE) was obtained by the following formulation:

$$\text{EE}\%\;=\;\frac{\text{the amount of baicalein in liposomes}}{\text{initial amount of baicalein for drug loading}}\times 100\%$$

### Cell Uptake of Baicalein-Loaded Liposomes

Cell uptake of baicalein-loaded liposomes in Hs68 human foreskin fibroblasts was examined using the fluorescent microscopy. DiI solution (1,1′-Dioctadecyl-3,3,3′,3′-Tetramethylindocarbocyanine Perchlorate, 10 mg/ml, 1 μl) was added into liposomal solution to form DiI-loaded liposomes. Hs68 fibroblasts were cultured in 6 cm dishes at a density of 3 × 10^5^ cells/ dish overnight at 37°C with 5% CO_2_. After 1 day culture, cell culture medium was removed and replaced with DiI-loaded liposomes at various time intervals (0–24 h) prior to washing with PBS and fixation with 0.075% (v/v) formaldehyde solution for 30 min. In the control group, Hs68 fibroblasts were treated with DiI solution for 24 h. After fixation, cells were secondary washed with PBS and stained nuclei using DAPI solution (10 μg/ml) for 10 min. Finally, Hs68 fibroblasts were rinsed and mounted with PBS and photographed by microscope (Nikon TI-E) and CCD camera system (SPOT RT3). Fluorescent photographs were quantitative analyzed using Image J software (NIH, United State). Cell uptake efficiency was determined via measuring DiI fluorescence intensity.

### Cell Viability

Human foreskin fibroblasts, Hs68 and murine macrophages, RAW264.7 were seeded at a density of 10^4^ cells/well and 5 × 10^4^ cells/well in 96 well plates individually for cell viability analysis. After incubation overnight, cells were then treated with pure baicalein, empty or baicalein-loaded liposomes in concentrations of 10 or 20 μg/ml respectively for 24 h. After baicalein treatment, cell culture medium were replaced with 200 μl of 100 μg/ml MTT (3-(4,5-Dimethylthiazol-2-yl)-2,5-diphenyltetrazolium bromide) reagent and incubated for 4 h before measuring absorbance at 570 nm using ELISA reader (Tecan, infinite M200). Relative cell viability was demonstrated by percentage compared with control.

### Nitrite Assay

RAW264.7 macrophages were seeded at a density of 5 × 10^5^ cells/well in 24 well plates, following by treatment with pure baicalein, empty or baicalein-loaded liposomes, in concentrations of 10 and 20 μg/ml respectively for 24 h. After removing of supernatants, no-phenol red medium (200 μl) were added for 8 h of incubation. The release of nitric oxide from inflamed macrophages was measured by determining nitrite concentration. Nitrite-contained medium (100 μl) were mixed with 100 μl of griess reagents in 96 well plates. Absorbance was measured at wavelength of 550 nm using ELISA reader (Tecan, infinite M200) after 15 min of shaking in dark. The reference values of nitric oxide were shown as the mean percentage of absorbance and standard deviation in comparison with lipopolysaccharide (LPS)-treated cells from two independent experiments.

### Oil Red O Staining

Hs68 fibroblasts were seeded at a density of 10^5^ cells/ dish in 6 cm dishes. After 24 h incubation, the culture medium was replenished with 20% (v/v) ADM and cultured for 14 days to induce lipogenesis, while pure baicalein, empty or baicalein-loaded liposomes were added at day 7. At the end of 14 days culture, cell culture medium was removed and rinsed with PBS twice. For histochemical examination, intracellular accumulation of lipid in Hs68 fibroblasts was performed using the Oil-red O staining kit (Lifeline Cell Technology, Carlsbad, CA, United States). Concisely, cells were fixed with 4% (v/v) paraformaldehyde fixative solution for 30 min, rinsed with PBS and incubated with 100% 1, 2-propanediol dehydration solution for 10 min at room temperature. Following by fixation and dehydration, oil red O stain solution was added and incubated at 37°C for 30 min prior to imaging of cell morphology under microscope (Nikon TI-E) and CCD camera system (SPOT RT3). Finally, 85% (v/v) 1,2-propanediol stain differential solution was added for 1 min to differentiate stain and the absorbance was measured by ELISA reader (Tecan, infinite M200) at the wavelength of 520 nm.

### Triglyceride Assay

Quantitative analysis of triglyceride content in ADM-induced Hs68 fibroblasts was conducted using an adipogenesis assay kit (Sigma–Aldrich) according to the manufacturer’s instructions. Hs68 fibroblasts were cultured and adipogenesis was induced as described above. Free drug, empty liposomes or baicalein-loaded liposomes were added to cells respectively at day 7. After 21 days of incubation, 100 μl of lipid extraction buffer was added and incubated for 30 min at 90°C. Cell medium became cloudy and then cooled down at room temperature. Cell medium was shaken for 1 min for homogenization followed by transfering 5–50 μl of lipid to another 96 well plate which were filled with adipogenesis assay buffer up to a total volume of 50 μl. Lipase solution (2 μl) was added and incubated for 10 min at room temperature for the degradation of triglyceride, following by adding master reaction mix for reaction of 30 min before measuring the absorbance at 570 nm using the ELISA reader (Tecan, infinite M200). Triglyceride content in Hs68 fibroblasts was calculated from the triglyceride (triolein)-equivalent standard curve.

### Fluorescent Antibody Technique

Hs68 fibroblasts were seeded at a density of 10^5^ cells/dish and treated with ADM for 14 days in the presence or absence of liposomal samples. After 14 days of adipogenic differentiation, medium was removed and rinsed with PBS. Cells were fixed with 10% formaldehyde at 4°C for 30 min and washed with PBS twice. 0.1% Nonidet P-40 in PBS was added and incubated at room temperature for 10 min. After discarding Nonidet P-40 solution, cells were washed with PBS twice and blocked with BSA solution (2% w/v in PBS) for 30 min. Thereafter, the primary antibodies of anti-collagen I IgG produced in rabbit (1:500 in PBS, ab34710, Abcam, Cambridge, United Kingdom), anti-collagen III IgG produced in mouse (1:1000 in PBS, ab6310, Abcam, Cambridge, United Kingdom), and anti-elastin IgG produced in rabbit (1:200 in PBS, ab21610, Abcam, Cambridge, United Kingdom) were added separately and kept at 4°C for overnight. After washing with PBS twice, the secondary antibody of H&L Dylight 594 anti-mouse IgG produced in goat (1:50 in PBS, ab96881 Abcam, Cambridge, United Kingdom) conjugated with reddish fluorescein (FITC) and green fluorescent anti-rabbit IgG produced in goat (1:200 in PBS, 111-095-003, Jackson ImmunoResearch Laboratories, WestGrove, PA, United States) were added in turn for 1 h of incubation. Finally, cells were washed with PBS twice and DAPI (10 μg/ml) was used to stain the nucleus of cells. The cell morphology was photographed by fluorescent microscopy equipped with CCD camera system (SPOT RT3). For quantitative analysis of elastin, type I and III collagen in hs68 fibroblasts, we used Image J software (NIH) to measure the fluorescence intensity in 10X images obtained with equal acquisition parameters.

### Collagen Assay

Collagen content in Hs68 fibroblasts was measured by Sircol collagen assay kit (Biocolor, United Kingdom) according to manufacturer’s instructions. Hs68 fibroblasts were seeded at a density of 10^5^ cells/ well in 6 well plates. Hs68 fibroblasts were incubated with 20% ADM for 7 days at 37°C in a 5% CO_2_ atmosphere and liposomal samples were added at day 3. At day 7, the medium was removed and washed with PBS. Pepsin solution (10 mg/ml in 0.5 M cold acetic acid) was added to each well for collagen isolation. After the supernatants were transferred to other eppendorf tubes, 1 ml Alkali Reagent was added and mixed by inverting contents for 30 min, then the samples were centrifuged at 12,000 × *g* for 10 min at 4°C. Afterward, the supernatant was removed and 750 μl ice-cold Acid-Salt Wash Reagent was added into the collagen-dye pellet to eliminate unbound dye from the surface of the pellet. After centrifuged at 12,000 × g for 10 min, the supernatants were removed and 250 μl Alkali Reagent was added to each sample to dissolve the dye. Finally, the absorbance was measure by an ELISA reader at a wavelength of 555 nm.

### Elastin Assay

Elastin content in Hs68 fibroblasts was measured by Fastin elastin assay kit (Biocolor, United Kingdom) according to manufacturer’s instructions. Firstly, Hs68 fibroblasts were seeded at a density of 10^5^ cells/ well in 6 well plates. Hs68 fibroblasts were incubated with 20% ADM for 7 days at 37°C in a 5% CO_2_ atmosphere, and test samples were treated to cells at day 3. At day 7, the medium was removed and washed with PBS. Then, 1 ml of trypsin solution was added into each well to detach Hs68 fibroblasts, and cell lysing solution was transferred to a 1.5 ml micro centrifuge tube. In order to convert cell bound elastin to water soluble α-elastin, 300 μl of cell lysing solution was mixed with 100 μl of 1.0 M oxalic acid to make the final concentration of 0.25 M and heated at 100°C for an hour. Afterward, an equal volume of elastin precipitating reagent was added and vortexed gently for 15 min to make sure the complete precipitation. After centrifugation at 10,000 g and 4°C for 10 min, the supernatant was discarded and replaced with 1ml of Dye Reagent. The samples were shaken evenly for 90 min and centrifuged at 10,000 *g* for another 10 min. The unbound dye was drained out and 250 μl of Dye Dissociation Reagent was added to each sample to dissolve the dye. Finally, the absorbance was measure by an ELISA reader at a wavelength of 513 nm.

### Quantitative Real-Time PCR

Hs68 fibroblasts were cultured in 6 cm dishes at different cell densities for the studies of TNF-α induction and ADM stimulation. After treatment, the total RNA in cells were extracted using Trizol reagent (Protech Technology, Taiwan) in reference to the manufacturer’s instructions. Messenger RNA was subsequently reverse transcribed to complementary DNA following the method of TProfessional basic (Biometra, Germany). The obtained cDNA was quantified to 20 ng and the measurement was conducted in StepOnePlus^TM^ Real-Time PCR system with FastStart DNA Mater-PLUS SYBR Green I (Applied Biosystem, United States). The primary sequences were shown in **Table 1**. The efficiency of DNA amplification was performed using the mean cycle threshold (Ct) method, which represent the number of cycles at which the fluorescence surpasses the threshold. ΔCt value in each group was calculated from Ct values of different genes subtracting the Ct value of GAPDH. The resulting relative mRNA expression was showed as fold change (2^-ΔΔCt^) relative to the expression values in control cells.

**Table 1:** Primer sequences used Real-Time PCR experiment.

Primer	Sequence (Forward) (5′→3′)	Sequence (Reverse) (5′→3′)
GAPDH	CATGAGAAGTATGACAACAGCCT	AGTCCTTCCACGATACCAAAGT
TNF-α	GACAAGGTGTACGTGAACATCG	CCACACTGTGTCGCCGTAG
IL-1β	AGCTACGAATCTCCGACCAC	CGTTATCCCATGTGTCGAAGAA
IL-6	TGGCTGAAAAAGATGGATGCT	TCTGCACAGCTCTGGCTTGT
IL-8	ACTGAGAGTGATTGAGAGTGGAC	AACCCTCTGCACCCAGTTTC
MMP-1	CTGGCCACAACTGCCAAATG	CTGTCCCTGAACAGCCCAGTACTT
MMP-3	ATTCCATGGAGCCAGGCTTTC	CATTTGGGTCAAACTCCAACTGTG
COX-2	GCCCTTCACGTTATTGCAGATG	ATATGTTCTCCTGCCTACTGGAA
LPL	GGACTTGGAGATGTGGACCA	TGCTGCTTCTTTTGGCTCTG
FABP4	AAAGTCAAGAGCACCATAACC	TTCAATGCGAACTTCAGTCC

### Statistical Analysis

Statistical analysis was completed by using commercial software Minitab 16. Data are presented as means ± standard deviations. All *p*-values were calculated by two-tails *t*-test and paired *t*-test. *P* < 0.05 was considered the significant difference.

## Results

### Characterization of Liposomal Formulations

**FIGURE 1:**
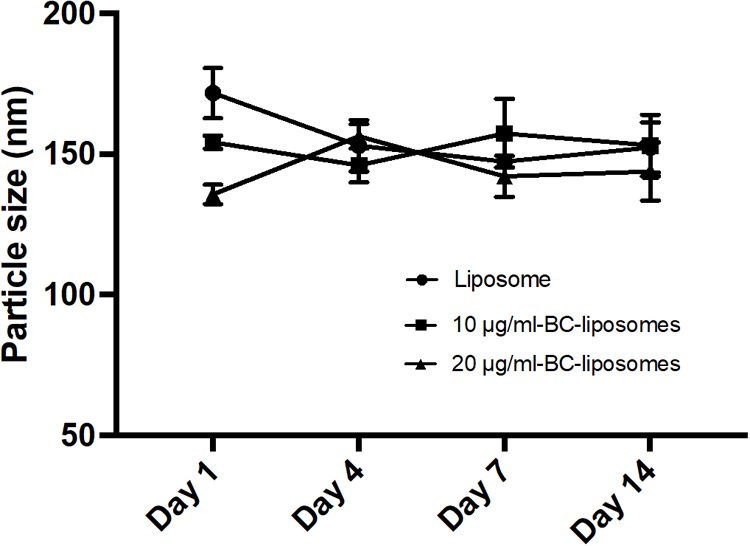
Stability of liposomal formulations. Empty and baicalein (BC)-loaded liposomes were stored at 4°C over different time intervals.

Physical property is an important parameter in formulation of liposomes as it could affect drug delivery or interaction between drugs and cells. In the present study, physical characteristics of baicalei-loaded liposomes, including particle size, entrapment efficiency, polydispersity index (PDI) and zeta potential, were assessed. **Table 2** illustrates that the particle sizes of empty liposomes, 10 μg/ml baicalein-loaded liposomes (10 μg/ml BC-Lip) and 20 μg/ml baicalein-loaded liposomes (20 μg/ml BC-Lip) are in the range of 135–171 nm. Liposomes encapsulated with baicalein were found with reduced particle size in a dose-dependent manner. Moreover, an increase of baicalein concentration in liposomes led to a decrease in PDI from 0.546 to 0.462. PDI is an important parameter which is used to describe variation of particle size in a population of particles and hence baicalein-loaded liposomes have a relatively narrow size distribution. Moreover, baicalein-loaded liposomes present a decline in entrapment efficiency from 33.65 to 25.40% when drug concentration increase from 30 to 80 μg/ml. The surface charge of baicalein-loaded liposomes was assessed through zeta potential measurement. Since baicalein-loaded liposomes are mainly composed with phosphatidylcholine, an amphoteric phospholipid, these liposomes should exhibit a neutral surface and hence the zeta potential was around zero. Similar values of zeta potential were obtained among these liposomal formulations, suggesting that the presence of baicalein did not alter the electrophoretic mobility of liposomes. The stability of liposomal formulations was further determined through particle size (**Figure 1**). Although particle sizes of empty and baicalein-loaded liposomes were slightly different at day 1, all the liposomes displayed similar stability profiles over a 2-week period at 4°C in the fridge. The stability of liposomes *in vivo* can also be affected by interactions between lipoproteins and few other proteins in the blood. In our previous serum stability studies of liposomes, no significant change in the particle size distribution for all the types of liposomal formulations was noted (Yeh et al., 2015).

**Table 2:** Physical parameters of the liposomal formulations after extrusion.

Drug formulation	Particle size (nm)	Entrapment efficiency (%)	PDI	Zeta potential (mV)
Empty liposomes	171.67 ± 8.92	—	0.546	-1.69
Baicalein-loaded liposomes (10 μg/ml)	154.17 ± 2.34	33.65%	0.503	-2.11
Baicalein-loaded liposomes (20 μg/ml)	135.67 ± 3.45	25.40%	0.462	-1.89

### Effect of Liposomal Formulations on the Cell Viability, Delivery Efficiency and NO Production

**FIGURE 2:**
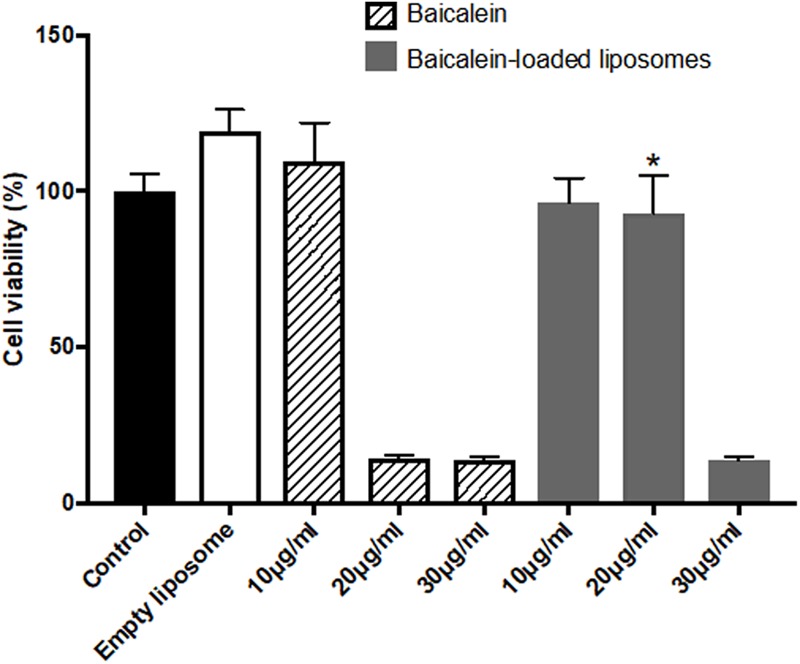
The effect of baicalein, empty and baicalein-loaded liposomes on cell viability of Hs68 fibroblasts. Cells were treated with different concentrations of baicalein and baicalein-loaded liposomes for 24 h and then measured by MTT assay. Cell viability of control was expressed as 100%. The data are presented as means ± standard deviation (*n* = 4–8). ^∗^*p* < 0.05 relative to 20 μg/ml Baicalein-treated cells.

The *in vitro* cytotoxic of free baicalein, empty and baicalein-loaded liposomes on Hs68 fibroblasts and RAW264.7 macrophages were examined using the MTT assay. As shown in **Figure 2**, free baicalein at the concentration of 20 μg/ml was found to reduce cell viability sharply to 14%. In comparison, liposome-encapsulated baicalein at the same concentration showed highly remained cell viability (around 100%), indicating that liposomal formulation was capable to prevent the cytotoxicity of baicalein with significantly enhanced cell viability. However, both free baicalein and liposome-encapsulated baicalein at the concentration of 30 μg/ml showed low cell viability (less than 15%).

**FIGURE 3:**
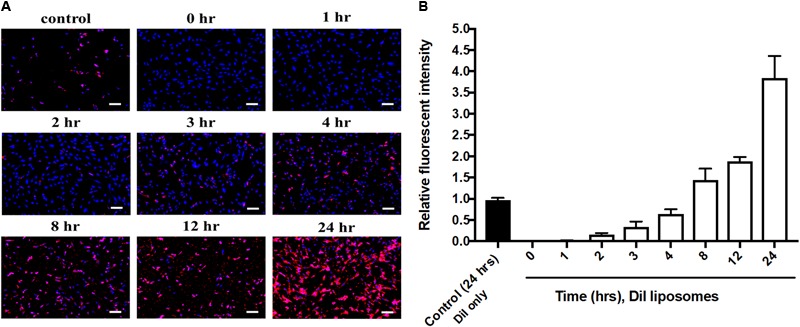
Cellular uptake of DiI-loaded liposomes in Hs68 fibroblasts. **(A)** Hs68 fibroblasts were incubated with DiI and DiI-loaded liposomes at different time intervals (1–24 h). The fluorescent image was photographed under fluorescent microscopy paired with CCD system. **(B)** The fluorescent intensity of DiI-loaded liposomes inside the cells was quantified using Image J. The data are expressed in relative index compared with control. The results are presented as the means ± standard deviation (×100 magnification, scale bar = 200 nm, *n* = 4).

Cell uptake of baicalein-loaded liposomes in Hs68 fibroblasts was analyzed based on release of DiI in to cells after cell uptake of DiI-labeled liposomes (Yeh et al., 2015). In here, DAPI stained cell nucleus apparent in blue fluorescence. As shown in **Figure 3A**, Dil-loaded liposomes were gradually taken by cells showing more red fluorescence signals over 24 h. Quantitative analysis of DiI red fluorescence signals demonstrated the fluorescent intensity in cells which were treated with DiI-loaded liposomes was four times higher compared to control cells which were treated with Dil only over 24 h (**Figure 3B**). Hence, liposomal formulation was helpful to improve cell viability and cellular uptake efficiency of baicalein in Hs68 fibroblasts.

**FIGURE 4:**
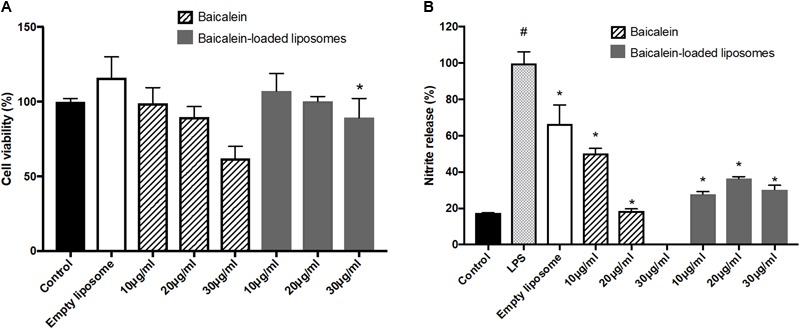
The effect of empty and baicalein-loaded liposomes on cell viability and nitrite production of RAW264.7 macrophages. **(A)** Cells were incubated with baicalein, empty and baicalein-loaded liposomes for 24 h, and then cell viability was measured using MTT assay. ^∗^*p* < 0.05 relative to 20 μg/ml Baicalein-treated cells **(B)** RAW264.7 macrophages were induced to inflammation by 500 ng/ml LPS. The inhibitory effect of baicalein and baicalein-loaded liposomes on nitrite production of LPS-induced RAW264.7 macrophages was determined after 24 h of incubation. The total nitrite production in LPS-stimulated cells is expressed as 100%. The data are presented as the means ± standard deviation. ^#^*P* < 0.05 relative to control and ^∗^*p* < 0.05 relative to LPS-stimulated cells (*n* = 3).

To confirm the anti-inflammatory activity of liposome-encapsulated baicalein, cell viability and nitrite production of RAW264.7 were measured. Liposome-encapsulated baicalein showed higher cell viability compared to free drug in treating RAW264.7 macrophages. Baicalein and liposome-encapsulated baicalein dose-dependently reduced cell viability of RAW264.7 macrophages (**Figure 4A**). Based on this result, concentrations with cell viability over 80% were selected for subsequent NO inhibition experiment. The nitrite accumulation in the cells was significantly increased after LPS stimulation. Therefore, cells were simultaneously treated with LPS following by free baicalein, empty or baicalein-loaded liposomes, respectively in order to determine anti-inflammatory activity of baicalein (**Figure 4B**). LPS-induced nitrite production in RAW264.7 macrophages was significantly inhibited through the treatment of free baicalein, empty or baicalein-loaded liposomes. Baicalein inhibited the NO production in a dose-dependent manner, particularly at 20 μg/ml of baicalein (greater than 80% inhibition). liposome-encapsulated baicalein also exhibited the suppressive effect on LPS-induced nitrite production (in approximate 65–75% inhibition), but not in a dose-dependent manner. The result of MTT assay confirmed that baicalein or liposome-encapsulated baicalein had direct anti-inflammatory effects which was not correlated with cell damage (cell viability > 80%).

### The Effect of Liposome-Encapsulated Baicalein on Lipid Accumulation and Triglyceride Synthesis in ADM-Induced Hs68 Fibroblasts

**FIGURE 5:**
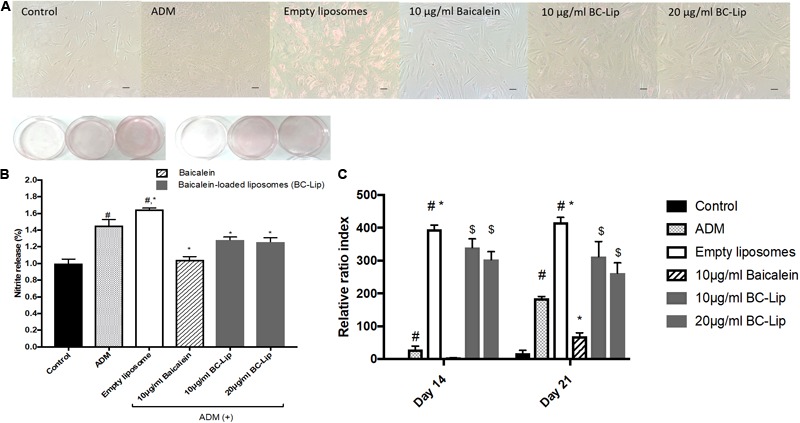
Lipid accumulation in human dermal fibroblasts, Hs68 **(A)** after the differentiation, lipid accumulation in the cells was stained with Oil Red O dye and visualized under a microscope at 100x of magnification. (Scale bar = 200 μm). **(B)** The quantification of lipid accumulation in Hs68 cells was measured using an ELISA reader at 500 nm. The data are presented as relative index to control, and the results were expressed as means ± standard deviation (*n* = 3–5). **(C)** Intracellular triglyceride content were determined with TG adipogenesis kit at 570 nm in Hs68 cells after 14 and 21 days of incubation with 20% ADM. The results were expressed by the mean intensity of triglyceride compared to control ± standard deviation (*n* = 3–8). ^#^*P* < 0.05 relative to control, ^∗^*p* < 0.05 relative to cells treated with ADM and ^$^*p* < 0.05 relative to cells co-treated with ADM and empty liposomes.

Lipogenesis effects of baicalein or liposome-encapsulated baicalein were examined respectively on Hs68 fibroblasts within ADM induced lipogenesis for 14 days. Oil Red-O was utilized to measure intracellular lipid accumulation. Under light microscopy, lipid droplets were apparent via treatments of ADM in the presence or absence of empty liposomes (**Figure 5A**). Moreover, cells co-treated with ADM and empty liposomes had a significantly increase in intracellular lipid content compared to the cells treated with ADM only. In contrast, baicalein (10 μg/ ml) showed a significant inhibitory role against ADM-induced lipid formation, while baicalein-loaded liposomes showed comparable inhibitory effects on lipid formation but not dose dependently (**Figure 5B**). These results were further confirmed by quantitative analysis of intracellular triglyceride contents. Suppressive effect on triglyceride accumulation was observed in Hs68 fibroblasts (**Figure 5C**). As expected, ADM induced triglyceride synthesis in Hs68 fibroblasts was significantly suppressed by free baicalein (10 μg/ml), while after liposome-encapsulation, baicalein-loaded liposomes were found to reduce ADM induced triglyceride synthesis, suggesting that a similar but mild effects of baicalein after liposome-encapsulation on the lipid formation and triglyceride synthesis in Hs68 fibroblasts.

### The Effect of Liposome-Encapsulated Baicalein on Gene Expressions of Lipogenesis Enzymes in ADM-Induced Hs68 Fibroblasts

**FIGURE 6:**
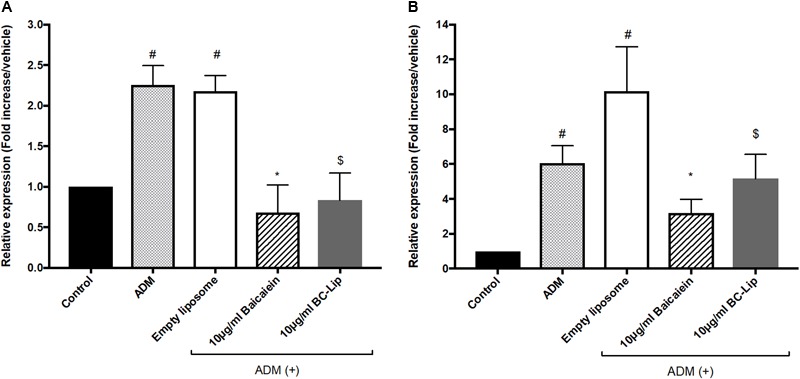
The effect of baicalein, empty and baicalein-loaded liposomes on adipogenic marker genes, LPL **(A)** and FABP4 **(B)** in the presence of ADM. The mRNA expressions of LPL and FABP4 were measured by real-time PCR analysis after 3 days incubation with ADM. Levels of LPL and FABP4 mRNA expression are presented relative to control gene expression. The data were presented as the mean ± standard deviation (*n* = 3). ^#^*P* < 0.05 relative to control, ^∗^*p* < 0.05 relative to cells treated with ADM only and ^$^*p* < 0.05 relative to cells co-treated with ADM and empty liposomes.

To understand the molecular mechanism of baicalein’s effects with or not encapsulated in liposomes on lipid accumulation in Hs68 cells, we examined the expression of lipogenesis enzymes, lipoprotein lipase (LPL) and fatty acid binding protein 4 (FABP4). A previous study demonstrated that the gene expressions of LPL and FABP4 in human adipose-derived stromal cells were significantly increased during adipogenic differentiation (D7–D21) and hence they could be served as potential adipogenic differentiation markers (Ambele et al., 2016). Cells treated with ADM, in presence or absence of empty liposomes, resulting in greater gene expression of LPL (2 fold) and FABP4 (sixfold) comparing to control and hence empty liposomes had no additive effect on lipogenesis (**Figure 6**). The increase of these lipogenic enzyme genes indicates that Hs68 fibroblasts can be induced to lipogenesis by ADM treatment no matter the addition of liposomes. In contrast to ADM-induced lipogenesis in Hs68 fibroblasts, free baicalein treatment demonstrated 70 and 48% decrease in LPL and FABP4 expression levels, respectively. Cells co-treated with ADM and liposome-encapsulated baicalein also exhibited suppressive effects on LPL (62% decrease) and FABP4 (49% decrease) mRNA expressions. Such expression profiles of LPL and FABP4 are consistent with lipid and triglyceride levels. Taken together, both free baicalein and liposome-encapsulated baicalein inhibited lipid formation and triglyceride synthesis in Hs68 fibroblasts through suppression of lipogenic enzyme genes.

### The Effect of Liposome-Encapsulated Baicalein on Gene Expression of Inflammation-Related Factor After ADM Treatment

**FIGURE 7:**
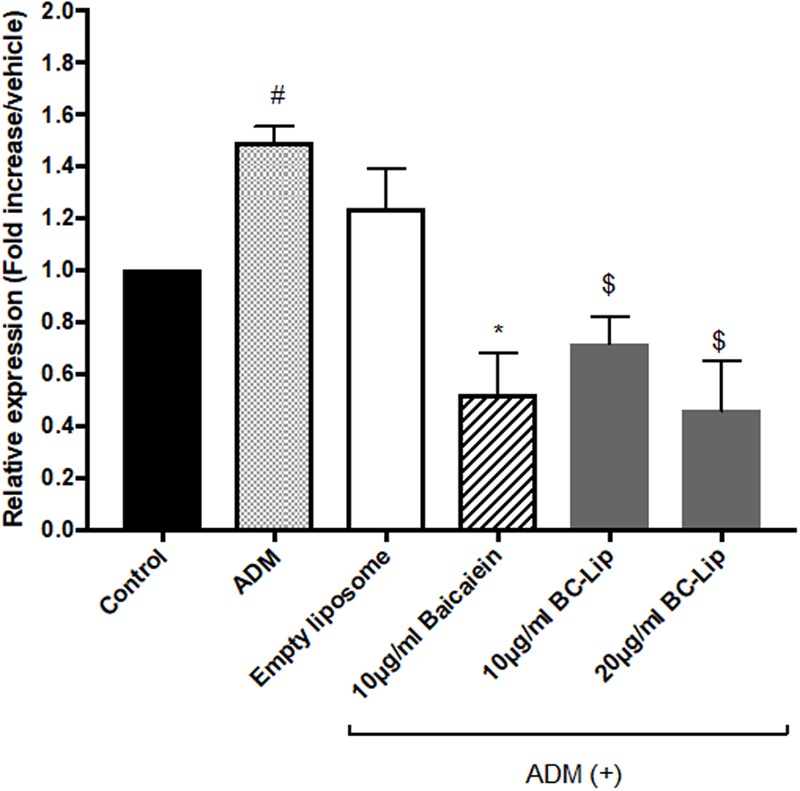
The effect of ADM, Baicalein and baicalein-loaded liposomes on gene expression of TNF-α. The level of TNF-α mRNA expression was measured using RT-PCR. The level of TNF-α mRNA expression is presented relative to control gene expression. The data were presented as the mean ± standard deviation (*n* = 3). ^#^*P* < 0.05 relative to control, ^∗^*p* < 0.05 relative to cells treated with ADM only and ^$^*p* < 0.05 relative to cells co-treated with ADM and empty liposomes.

As obesity is known to induce mild inflammatory responses, while inflammation plays an important role in insulin resistance, diabetes and other diseases. Hotamisligil reported that the mRNA expression of tumor necrosis factor alpha (TNF-α) mRNA expression in subcutaneous adipose tissue were about 2.5-fold higher in obese women compared to women with normal BMI. Importantly, reduction of body weight (17% w/w) were found to correlate with 22% decrease of TNF-α expression, and lead to indirectly improvement of insulin sensitivity (Hotamisligil et al., 1995). Thus, TNF-α expression was used in the present study as an indicator to determine the release of adipokines in the adipose tissue (Cawthorn and Sethi, 2008). To clarify whether ADM-induced adipogenesis can stimulate inflammation in Hs68 fibroblasts, we measured the mRNA levels of TNF-α. In here, we found that ADM treatment resulted in 1.5-fold increase in the gene expression of TNF-α, and which can be reduced approximate 75 and 80% by baicalein and liposome-encapsulated baicalein respectively (**Figure 7**). In addition, ADM-induced TNF-α expression can be suppressed by liposome-encapsulated baicalein in a dose-dependent manner. A previous report using fresh peritoneal murine macrophages harvested from C57RL/6 mice or ANA-1 macrophage line derived from the bone marrow of C57RL/6 mice demonstrated that LPL can induce TNF-α gene expression and protein secretion (Renier et al., 1994). Moreover, Xu et al. (2015) also indicated that FABP4 could modulate TNF-α secretion in FABP4/aP2 knockout macrophages, suggesting ADM treatment may induce TNF-α expression in Hs68 fibroblasts through the up-regulation of lipogenic enzyme gene expressions (LPL and FABP4).

**FIGURE 8:**
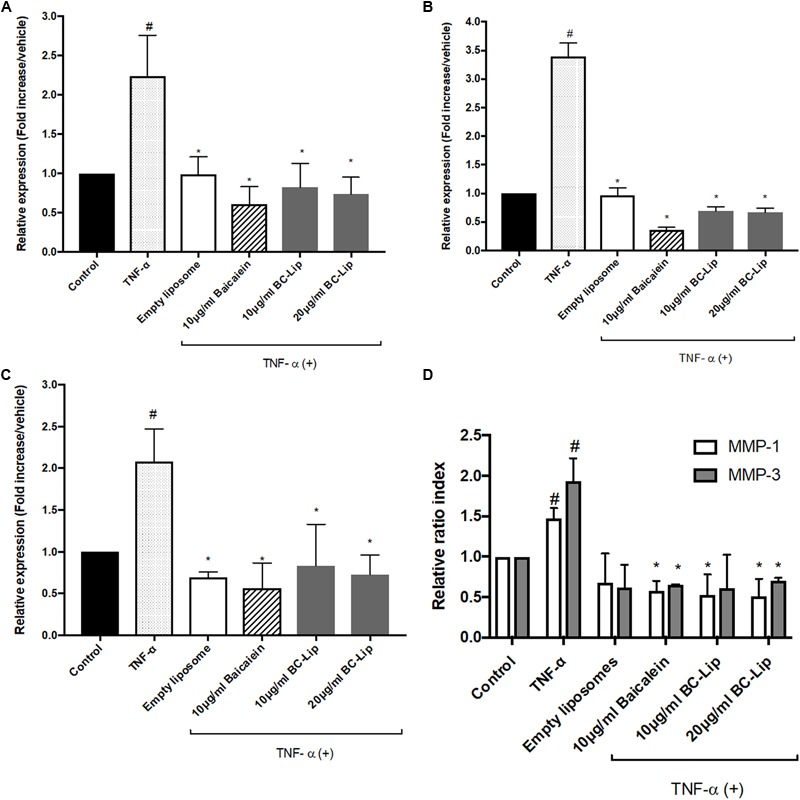
The effect of baicalein, empty and baicalein-loaded liposomes on TNF-α-induced inflammatory responses in Hs68 fibroblasts. Hs68 fibroblasts were treated with test samples in the presence or absence of TNF-α for an hour. Afterward, the gene expressions of COX-2 **(A)**, IL-6 **(B)** and IL-8 **(C)** were determined by real-time PCR. **(D)** The mRNA expressions of MMP-1 and MMP-3 were also measured after 4 h incubation with test samples in the presence or absence of TNF-α. Levels of COX-2, IL-6, IL-8, MMP-1 and MMP-3 mRNA expression are presented relative to control gene expression. The results were expressed as the mean ± standard deviation (*n* = 3). ^#^*P* < 0.05 relative to control and ^∗^*p* < 0.05 relative to cells treated with TNF-α only.

ADM-induced adipogenesis caused inflammatory responses in Hs68 fibroblasts through the up-regulation of TNF-α gene expression. We further treated Hs68 fibroblasts treated with 20 ng/ml of TNF-α following by measure of the mRNA expressions of inflammatory cytokines, such as cyclooxygenase-2 (COX-2), interleukin-6 (IL-6) and IL-8. As shown in **Figures 8A–C**, incubation of Hs68 fibroblasts with TNF-α increased the gene expressions of COX-2, IL-6 and IL-8 (about 2, 2 and 3.5-fold) and the addition of baicalein, empty and baicalein-loaded liposomes individually down-regulated TNF-α-induced inflammatory gene expressions. Since TNF-α was reported to stimulate ECM degradation by inducing the expression of matrix metalloproteases (MMP)-1 and MMP-3 in dermal fibroblasts (Shindo et al., 2014), we then examined the effect of baicalein, empty and baicalein-loaded liposomes on gene expressions of MMP-1 and MMP-3. Incubation of Hs68 fibroblasts with TNF-α significantly increased gene expressions of MMP-1 and MMP-3 compared to non-treated control cells, whereas baicalein and liposome-encapsulated baicalein significantly reduced TNF-α-induced MMP-1 and MMP-3 mRNA levels (**Figure 8D**). It is interesting to note that empty liposomes have similar inhibitory effect on TNF-α-induced MMP-1 and MMP-3 mRNA expressions but not in statistical significance. Through the up-regulation of TNF-α gene expression, ADM-induced lipogenesis may cause inflammation and ECM degradation in Hs68 fibroblasts. However, baicalein, empty and baicalein-loaded liposomes show their potential to suppress inflammatory responses and MMP expressions via down-regulation of TNF-α pathway.

### The Effect of Liposome-Encapsulated Baicalein on ECM Synthesis in ADM-Induced Hs68 Fibroblasts

**FIGURE 9:**
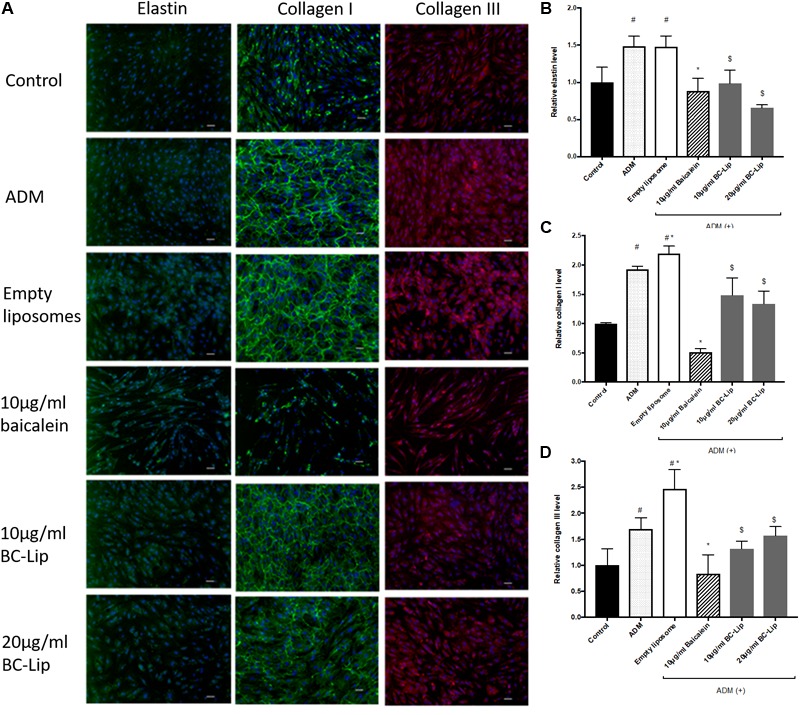
The effect of ADM, baicalein and baicalein-loaded liposomes on elastin, type I and type III collagen protein expressions in Hs68 fibroblasts. Immunofluorescence staining was carried out using specific antibody. **(A)** The immunofluorescent image of elastin, type I and type III collagen were photographed under microscopy paired with CCD system (×100 magnification, Scale bar = 200 μm). Quantification of fluorescent intensity for protein expression levels of **(B)** elastin, **(C)** type I collagen and **(D)** type III collagen was determined by Image J. The levels of protein expression are presented relative to control. The results were expressed as the mean ± standard deviation (*n* = 3). ^#^*P* < 0.05 relative to control, ^∗^*p* < 0.05 relative to cells treated with ADM and ^$^*p* < 0.05 relative to cells co-treated with ADM and empty liposomes.

Dermal fibroblasts have major responsibility to produce ECM for maintaining skin homeostasis and for orchestrating skin tissue regeneration. Since ECM plays a critical role in regulation of skin cell morphogenesis, activity and function, ADM, baicalein and liposome-encapsulated baicalein was further investigated on ECM synthesis. In the presence of ADM and empty liposomes, Hs68 fibroblasts were found enlarged and altered in cell shape. In addition, cells co-treated with ADM and empty (or baicalein-loaded) liposomes produced an elaborate type I collagen matrix (**Figure 9A**), whereas control cells or cells co-treated with ADM and baicalein showed no cross-linked network of collagen microfibers. Quantitative analysis of the fluorescent signals revealed that cells treated by ADM or empty liposomes exhibited higher expression levels of elastin, type I and type III collagens compared to control and cells co-treated with ADM and baicalein (or liposome-encapsulated baicalein) (**Figures 9B–D**). These results indicated that baicalein (or liposome-encapsulated baicalein) can largely reduce the stimulatory effect of ADM and liposomal formulation on ECM synthesis.

**FIGURE 10:**
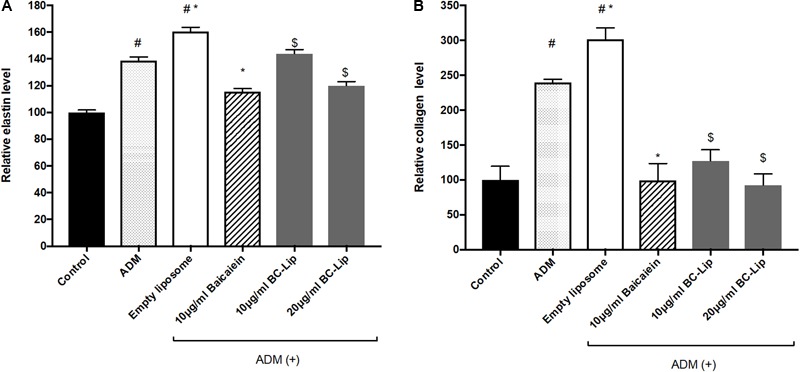
Protein expressions of elastin **(A)** and collagen **(B)** were measured by ELISA. The protein level was presented in relative proportion compared with control. The data were expressed as the mean ± standard deviation (*n* = 4). ^#^*P* < 0.05 relative to control, ^∗^*p* < 0.05 relative to cells treated with ADM only and ^$^*p* < 0.05 relative to cells co-treated with ADM and empty liposomes.

To further confirm the effect of baicalein and liposome-encapsulated baicalein, collagen and elastin protein level in Hs68 fibroblasts were analyzed using ELISA assay. Results are in agreement with immunohistochemical staining showing a similar declining trend in collagen and elastin production (**Figure 10**). Taken together, ADM and empty liposomes could induce ECM expression level in Hs68 fibroblasts but baicalein or liposome-encapsulated baicalein may have the reverse effects.

## Discussion

Previous studies revealed that baicalein is capable of attenuating ROS generation and exhibit high anti-oxidant activity (Chang et al., 2011). We also found that baicalein displays strong DPPH radical scavenging action, particularly at the concentration of 20 μg/ml (around 90%, **Supplementary Figure S1**). In a study reported by Kimura and Sumiyoshi (2011) baicalein could inhibit UV-B-induced MMP-9 and VEGF expression level through suppression of COX-2 but expression of NF-κB and hence baicalein is believed to have a remarkable anti-inflammation activity. In the present study, our findings demonstrate that baicalein is capable to decrease LPS-induced nitrite production in RAW264.7 macrophages and TNF-α stimulated COX-2, IL-6 and IL-8 expressions in Hs68 fibroblasts. These results agree with previous studies, showing that baicalein possesses excellent anti-inflammatory effect (Kimura and Sumiyoshi, 2011; Chen et al., 2014). However, due to its low solubility in aqueous solutions and low bioavailability *in vivo*, medical application of baicalein are very limited, particularly for skin administration. Few recent studies suggested that the therapeutic efficiency of natural compounds could be improved by liposomal nanoencapsulation because of its high compatibility and easy incorporation efficiency (Cadena et al., 2013; Caddeo et al., 2014). Encapsulated drugs in nanoparticles are known to reduce drugs leakage, prolong the residence time on skin and facilitate the internalization of drugs into cells (Moulaoui et al., 2015). Tsai et al. (2012) found that encapsulated baicalein with nanostructured lipid carrier (NLC) system can be used in brain therapy. In the present study, we used soybean phosphatidylcholine (SPC) for baicalein-loaded liposomal formulations to enhance drug bioavailability. Notably, particle size and PDI were decreased with baicalein–loaded liposomes, and no significant differences in zeta potential was measured between empty liposomes and baicalein-loaded liposomes. The reduction of particle size may be due to stronger drug interactions via hydrogen bonding and hence there is no effect on zeta potential. After 14 days stored at 4°C, only less 20% variation in particle sizes was noted for empty or baicalein-loaded liposomes. Moreover, our previous studies of liposome’ stability in serum demonstrated no significant change in the particle size distribution for all the liposomal formulations (Yeh et al., 2015). Hence, liposomal formulations are in high uniformity and homogeneity based on the low PDI and great stability within 14 days. In comparison with free drug, liposome-encapsulated baicalein (20 μg/ml, equal to 74 μM) obtained high cell viability in Hs68 fibroblasts due to the reduction of drug leakage and the anti-inflammatory activity of liposome-encapsulated baicalein is consistent with free drug.

Rakar et al. (2012) showed that primary human dermal fibroblasts were able to differentiate toward adipocytes, osteoblasts and chondrocytes using different induction media. Additionally, Takeda et al. (2017) also observed dramatic morphological change of human dermal fibroblasts over differentiation in which cells morphology convert from characteristic elongated fibroblasts into round adipocyte-like cells. In here, ADM-induced lipogenesis enlarged the cell size of Hs68 fibroblasts with oval and round cells rather than spindle shape. Such morphological changes of Hs68 human dermal fibroblasts during lipogenesis is consistent with the “morphotypes” described in previous studies (Rakar et al., 2012; Takeda et al., 2017). Conversely, Lu et al. (2006) previously demonstrated that baicalein at concentrations of 160–640 μM inhibited the proliferation of porcine preadipocytes with over expression of adipogenesis related genes: PPARγ2 and fatty acid synthase (FAS) but the gene expression was suppressed in lower concentrations of baicalein at 40–320 μM. Similarly, the inhibitory role of baicalein on lipid accumulation in 3T3-L1 fibroblasts and zebrafish was published by Seo et al. (2014). Baicalein inhibited triglyceride accumulation during adipogenesis and significantly down-regulated the gene expression of lipogenesis enzyme, FABP (Cha et al., 2006). In our study, as expected, baicalein or liposome-encapsulated baicalein remained cells with fibroblast-like morphology rather than to adipocytes, and reduced lipid droplet formation in ADM-induced Hs68 fibroblasts. Furthermore, Baicalein and liposome-encapsulated baicalein showed significant inhibitory effect on ADM-induced lipid accumulation and triglyceride synthesis through the suppression of adipogenesis marker, FABP4 and LPL. Thus, we believed that baicalein and liposome-encapsulated baicalein could suppress ADM-induced lipogenesis in human dermal fibroblasts.

As lipogenesis is known to induce inflammatory responses via the increase of TNF-α expression (Hotamisligil et al., 1995; Cawthorn and Sethi, 2008), ADM-induced lipogenesis markedly increased TNF-α expression in Hs68 fibroblasts through the up-regulation of LPL and FABP4 expression, and which was further inhibited by the addition of baicalein, empty and baicalein-loaded liposomes (Renier et al., 1994; Xu et al., 2015). Of note, baicalein, empty liposomes and baicalein-loaded liposomes demonstrated prominent suppression in COX-2, IL-1, IL-6, and IL-8 gene expressions in TNF-α induced Hs68 fibroblasts. These findings are in agreement with previous studies (Hsieh et al., 2007; Kimura and Sumiyoshi, 2011; Luo et al., 2017), showing baicalein can consistently suppress gene and protein expressions of Cox-2, IL-1, IL-6 and IL-8. Results herein further confirm that liposomes didn’t effect on inhibitory role of baicalein on gene expressions of inflammatory responses. Moreover, our results showed that pure baicalein and baicalein-loaded liposomes suppressed gene expression of MMP-1 and MMP-3, while similar results were reported before by Chen et al. (2015) through measuring the gene and protein expressions of MMP-1, 3, and 13 in baicalein treated human OA chondrocytes. They found baicalein can dose-dependently reduce gene and protein expressions of MMP1, 3 and 13 in IL-1β-induced human OA chondrocytes (Chen et al., 2015), while other researchers also demonstrated that baicalein can down-regulate gene and protein expressions of MMP-1 in H_2_O_2_-treated human HaCaT keratinocytes (Kim et al., 2012). According to our data, baicalein and baicalein-loaded liposomes have similar inhibitory effect on gene expressions of MMP-1 and MMP-3 and that is consistent with previous findings (Kim et al., 2012; Chen et al., 2015). Besides, baicalein inhibited the gene and protein expressions of MMP-2 and MMP-9 and meanwhile promoted the expressions of tissue inhibitors of metalloproteinase (TIMP)-1 and TIMP-2 in hepatocellular carcinoma MHCC97H cells and mouse melanoma B16F10 cells (Chen et al., 2013; Choi et al., 2017), suggesting that baicalein can suppress inflammatory responses through the down-regulation of TNF-α expression, resulting in the inhibition of MMP expressions in Hs68 fibroblasts.

Since ECM is playing a key role in skin regeneration, we therefore examined protein expressions of elastin, type I and type III collagens in Hs68 fibroblasts. Our results indicated that ADM stimulation up-regulated elastin, type I and type III collagens in Hs68 fibroblasts. Of note, liposomes slightly increased type I and type III collagens but not elastin as compared with ADM-treated cells. Similar to our findings, human subcutaneous adipose-derived cells were found to have presence of COL1A1 gene (Mariman and Wang, 2010). In a DNA microarray analysis of subcutaneous adipose tissue (SAT) and visceral adipose tissue (VAT) in Wistar rats, ECM-related genes such as type I, III, and V collagen were expressed higher in SAT than VAT (Mori et al., 2014). Moreover, collagen type I protein was highly expressed and formed a fibrous structure in rat SAT as well as dermis but not in rat VAT. In comparison with undifferentiated cells, a decrease of type I, type III and type V collagens was observed in 3T3-L1 cells during early phase of adipogenic differentiation (Wang et al., 2004; Mori et al., 2014). Enlarged adipocytes reduced 3T3-L1 fibroblast proliferation and gene expression of collagen type I and elastin and increased gene expression of MMP 13 (Ezure and Amano, 2011). Therefore, Hs68 fibroblast may present similar ECM expressions (such as type I and type III collagens) to human subcutaneous adipose-derived cells, rat SAT and VAT but not mouse 3T3-L1 adipocytes. Besides, baicalein and liposome-encapsulated baicalein suppressed the protein expressions of elastin, type I and type III collagen in ADM-induced Hs68 fibroblasts which may due to the inhibition of lipogenesis. Therefore, we elucidated that lipogenesis in Hs68 fibroblasts can increase ECM formulation, particularly in the protein expression of elastin, type I and III collagens. It is worth noting that baicalein had inhibitory effect on ECM formation in lipid-containing fibroblasts.

**FIGURE 11:**
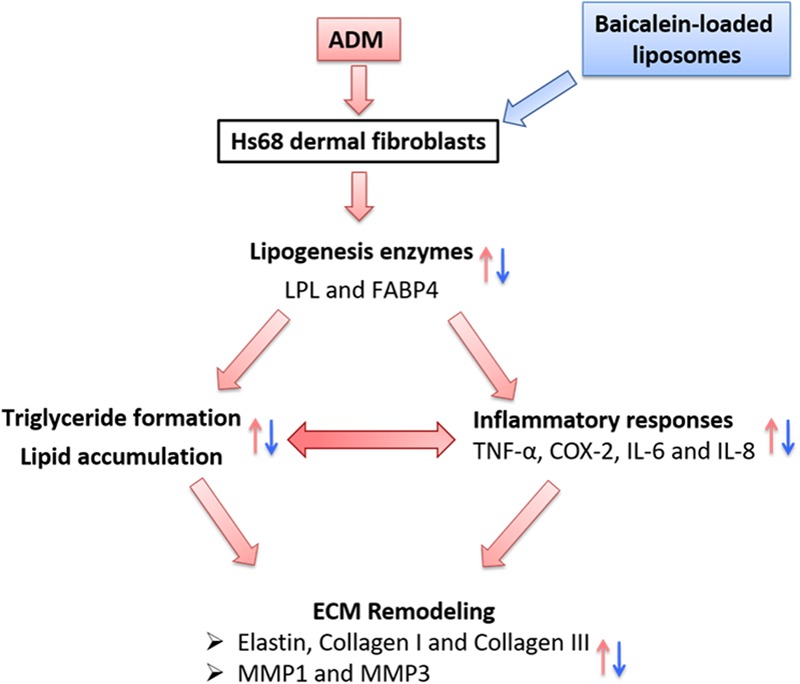
The hypothetic model of how adipogenesis, inflammation and ECM remodeling are regulated in Hs68 human dermal fibroblasts. Physiological stimuli such as ADM may signal through LPL or FABP4 to activate adipogenic and inflammatory pathways, which may be responsible for the upregulation of ECM remodeling.

In summary, we propose the following network: Physiological stimuli (ADM treatment) influenced the expressions of lipogenic enzyme genes (LPL and FABP4); simultaneously, lipogenesis enzymes control the lipid accumulation and inflammatory responses, which are the key factors for ECM remodeling in Hs68 fibroblasts (**Figure 11**). Besides, Liposome-encapsulated baicalein provide enhanced cell viability and cellular uptake efficiency of Hs68 fibroblasts together with down-regulation of ADM-induced lipid accumulation and ECM formation in Hs68 fibroblasts through suppression of lipogenesis enzymes and inflammatory responses. In conclusion, we suggested that liposome-encapsulated baicalein can provide an opportunity as medical or cosmetic products to prevent lipogenesis and maintain ECM structure in skin.

## Author Contributions

C-LF designed and performed the experiments and analyzed the data. KT performed the experiments. YW wrote the manuscript and contributed to data analysis. H-IC supervised the project, contributed to data analysis, and wrote the manuscript.

## Conflict of Interest Statement

The authors declare that the research was conducted in the absence of any commercial or financial relationships that could be construed as a potential conflict of interest. The reviewer AP and handling Editor declared their shared affiliation.

## Supplementary Material

FIGURE S1 The anti-oxidant activity of baicalein measured by DPPH scavenging assay.

## References

[B1] Ambele M. A. Dessels C. Durandt C. Pepper M. S. (2016). Genome-wide analysis of gene expression during adipogenesis in human adipose-derived stromal cells reveals novel patterns of gene expression during adipocyte differentiation. *Stem Cell Res.* 16 725–734. 10.1016/j.scr.2016.04.011 27108396

[B2] Bergstrand N. Arfvidsson M. C. Kim J. M. Thompson D. H. Edwards K. (2003). Interactions between pH-sensitive liposomes and model membranes. *Biophys. Chem.* 104 361–79. 10.1016/S0301-4622(03)00011-5 12834854

[B3] Bhattacharya M. (2016). Mesenchymal metamorphosis. *Sci. Transl. Med.* 8:370ec202. 10.1126/scitranslmed.aal3700 28003541

[B4] Caddeo C. Díez-Sales O. Pons R. Fernàndez-Busquets X. Fadda A. M. Manconi M. (2014). Topical anti-inflammatory potential of quercetin in lipid-based nanosystems: in vivo and in vitro evaluation. *Pharm. Res.* 31 959–968. 10.1007/s11095-013-1215-1210 24297068

[B5] Cadena P. G. Pereira M. A. Cordeiro R. B. Cavalcanti I. M. Neto B. B. Maria do Carmo C. B. (2013). Nanoencapsulation of quercetin and resveratrol into elastic liposomes. *Biochimica et Biophysica Acta* 1828 309–316. 10.1016/j.bbamem.2012.10.022 23103506

[B6] Cawthorn W. P. Sethi J. K. (2008). TNF-alpha and adipocyte biology. *FEBS Lett.* 582 117–131. 10.1016/j.febslet.2007.11.051 18037376 PMC4304634

[B7] Cha M. H. Kim I. C. Lee B. H. Yoon Y. (2006). Baicalein inhibits adipocyte differentiation by enhancing COX-2 expression. *J. Med. Food* 9 145–153. 10.1089/jmf.2006.9.145 16822198

[B8] Chang W. T. Li J. Haung H. H. Liu H. Han M. Ramachandran S. (2011). Baicalein protects against doxorubicin-induced cardiotoxicity by attenuation of mitochondrial oxidant injury and JNK activation. *J. Cell. Biochem.* 112 2873–2881. 10.1002/jcb.23201 21618589 PMC3178681

[B9] Chen K. Zhang S. Ji Y. Li J. An P. Ren H. (2013). Baicalein inhibits the invasion and metastatic capabilities of hepatocellular carcinoma cells via down-regulation of the ERK pathway. *PLoS One* 8:e72927. 10.1371/journal.pone.0072927 24039823 PMC3765161

[B10] Chen S. Yang Y. Feng H. Wang H. Zhao R. Liu H. (2014). Baicalein inhibits interleukin-1β-induced proliferation of human rheumatoid arthritis fibroblast-like synoviocytes. *Inflammation* 37 163–169. 10.1007/s10753-013-9725-9729 24005900

[B11] Chen W. P. Xiong Y. Hu P. F. Bao J. P. Wu L. D. (2015). Baicalein inhibits MMPs expression via a MAPK-dependent mechanism in chondrocytes. *Cell Physiol. Biochem.* 36 325–333. 10.1159/000374075 25967971

[B12] Choi E. O. Cho E. J. Jeong J. W. Park C. Hong S. H. Hwang H. J. (2017). Baicalein inhibits the migration and invasion of B16F10 mouse melanoma cells through inactivation of the PI3K/Akt signaling pathway. *Biomol. Ther.* 25 213–221. 10.4062/biomolther.2016.094 27530117 PMC5340547

[B13] de Oliveira M. R. Nabavi S. F. Habtemariam S. Erdogan Orhan I. Daglia M. Nabavi S. M. (2015). The effects of baicalein and baicalin on mitochondrial function and dynamics: a review. *Pharmacol. Res.* 100 296–308. 10.1016/j.phrs.2015.08.021 26318266

[B14] Ezure T. Amano S. (2010). Increased subcutaneous adipose tissue impairs dermal function in diet-induced obese mice. *Exp. Dermatol.* 19 878–882. 10.1111/j.1600-0625.2009.00970.x 19758317

[B15] Ezure T. Amano S. (2011). Negative regulation of dermal fibroblasts by enlarged adipocytes through release of free fatty acids. *J. Invest. Dermatol.* 131 2004–2009. 10.1038/jid.2011.145 21697886

[B16] Fang C. L. Huang L. H. Tsai H. Y. Chang H. I. (2016). Dermal lipogenesis inhibits adiponectin production in human dermal fibroblasts while exogenous adiponectin administration prevents against UVA-induced dermal matrix degradation in human skin. *Int. J. Mol. Sci.* 17:E1129. 10.3390/ijms17071129 27428951 PMC4964503

[B17] Galović Rengel R. Barisić K. Pavelić Z. Zanić Grubisić T. Cepelak I. Filipović-Grcić J. (2002). High efficiency entrapment of superoxide dismutase into mucoadhesive chitosan-coated liposomes. *Eur. J. Pharm. Sci.* 15 441–448. 10.1016/S0928-0987(02)00030-1 12036721

[B18] Gao Y. Lu J. Zhang Y. Chen Y. Gu Z. Jiang X. (2013). Baicalein attenuates bleomycin-induced pulmonary fibrosis in rats through inhibition of miR-21. *Pulm. Pharmacol. Ther.* 26 649–654. 10.1016/j.pupt.2013.03.006 23523661

[B19] Hew J. Solon-Biet S. M. McMahon A. C. Ruohonen K. Raubenheimer D. Ballard J. W. (2016). The effects of dietary macronutrient balance on skin structure in aging male and female mice. *PLoS One* 11:e0166175. 10.1371/journal.pone.0166175 27832138 PMC5104383

[B20] Hotamisligil G. S. Arner P. Caro J. F. Atkinson R. L. Spiegelman B. M. (1995). Increased adipose tissue expression of tumor necrosis factor-alpha in human obesity and insulin resistance. *J. Clin. Invest.* 95 2409–2415. 10.1172/JCI117936 7738205 PMC295872

[B21] Hsieh C. J. Hall K. Ha T. Li C. Krishnaswamy G. Chi D. S. (2007). Baicalein inhibits IL-1beta- and TNF-alpha-induced inflammatory cytokine production from human mast cells via regulation of the NF-kappaB pathway. *Clin. Mol. Allergy* 5:5. 10.1186/1476-7961-5-5 PMC2206049 18039391

[B22] Huang Y. Zhang B. Gao Y. Zhang J. Shi L. (2014). Baicalein-nicotinamide cocrystal with enhanced solubility, dissolution, and oral bioavailability. *J. Pharm. Sci.* 103 2330–2337. 10.1002/jps.24048 24903146

[B23] Kalepu S. Nekkanti V. (2015). Insoluble drug delivery strategies: review of recent advances and business prospects. *Acta Pharm. Sin. B* 5 442–453. 10.1016/j.apsb.2015.07.003 26579474 PMC4629443

[B24] Kim K. C. Kang S. S. Lee J. Park D. Hyun J. W. (2012). Baicalein attenuates oxidative stress-induced expression of matrix metalloproteinase-1 by regulating the ERK/JNK/AP-1 pathway in human keratinocytes. *Biomol. Ther.* 20 57–61. 10.4062/biomolther.2012.20.1.057 24116275 PMC3792202

[B25] Kimura Y. Sumiyoshi M. (2011). Effects of baicalein and wogonin isolated from *Scutellaria baicalensis* roots on skin damage in acute UVB-irradiated hairless mice. *Eur. J. Pharmacol.* 661 124–132. 10.1016/j.ejphar.2011.04.033 21549115

[B26] Koumas L. Smith T. J. Feldon S. Blumberg N. Phipps R. P. (2003). Thy-1 expression in human fibroblast subsets defines myofibroblastic or lipofibroblastic phenotypes. *Am. J. Pathol.* 163 1291–1300. 10.1016/S0002-9440(10)63488-8 14507638 PMC1868289

[B27] Li X. Luo W. Ng T. W. Leung P. C. Zhang C. Leung K. C. (2017). Nanoparticle-encapsulated baicalein markedly modulates pro-inflammatory response in gingival epithelial cells. *Nanoscale* 9 12897–12907. 10.1039/c7nr02546g 28650029

[B28] Li Y. Zhang C. Liu L. Gong Y. Xie Y. Cao Y. (2017). The effects of baicalein or baicalin on the colloidal stability of ZnO nanoparticles (NPs) and toxicity of NPs to Caco-2 cells. *Toxicol. Mech. Methods* 28 167–176. 10.1080/15376516.2017.1376023 28868948

[B29] Lu R. H. Li Y. Zhang L. J. Yang G. S. (2006). Effects of baicalein on the proliferation and differentiation of pig preadipocyte. *Chin. J. Biotechnol.* 22 1002–1006. 10.1016/S1872-2075(06)60069-1 17168327

[B30] Luo X. Yu Z. Deng C. Zhang J. Ren G. Sun A. (2017). Baicalein ameliorates TNBS-induced colitis by suppressing TLR4/MyD88 signaling cascade and NLRP3 inflammasome activation in mice. *Sci. Rep.* 7:16374. 10.1038/s41598-017-12562-6 29180692 PMC5703971

[B31] Mariman E. C. Wang P. (2010). Adipocyte extracellular matrix composition, dynamics and role in obesity. *Cell Mol. Life Sci.* 67 1277–1292. 10.1007/s00018-010-0263-4 20107860 PMC2839497

[B32] Mohammed A. R. Weston N. Coombes A. G. Fitzgerald M. Perrie Y. (2004). Liposome formulation of poorly water soluble drugs: optimisation of drug loading and ESEM analysis of stability. *Int. J. Pharm.* 285 23–34. 10.1016/j.ijpharm.2004.07.010 15488676

[B33] Mori S. Kiuchi S. Ouchi A. Hase T. Murase T. (2014). Characteristic expression of extracellular matrix in subcutaneous adipose tissue development and adipogenesis; comparison with visceral adipose tissue. *Int. J. Biol. Sci.* 10 825–833. 10.7150/ijbs.8672 25076859 PMC4115194

[B34] Moulaoui K. Caddeo C. Manca M. L. Castangia I. Valenti D. Escribano E. (2015). Identification and nanoentrapment of polyphenolic phytocomplex from *Fraxinus angustifolia*: in vitro and in vivo wound healing potential. *Eur. J. Med. Chem.* 89 179–188. 10.1016/j.ejmech.2014.10.047 25462238

[B35] Ong S. G. Ming L. C. Lee K. S. Yuen K. H. (2016). Influence of the encapsulation efficiency and size of liposome on the oral bioavailability of griseofulvin-loaded liposomes. *Pharmaceutics* 8:E25. 10.3390/pharmaceutics8030025 27571096 PMC5039444

[B36] Rajkumari J. Busi S. Vasu A. C. Reddy P. (2017). Facile green synthesis of baicalein fabricated gold nanoparticles and their antibiofilm activity against *Pseudomonas aeruginosa* PAO1. *Microb. Pathog.* 107 261–269. 10.1016/j.micpath.2017.03.044 28377235

[B37] Rakar J. Lönnqvist S. Sommar P. Junker J. Kratz G. (2012). Interpreted gene expression of human dermal fibroblasts after adipo-, chondro- and osteogenic phenotype shifts. *Differentiation* 84 305–313. 10.1016/j.diff.2012.08.003 23023066

[B38] Rehan V. K. Sugano S. Wang Y. Santos J. Romero S. Dasgupta C. (2006). Evidence for the presence of lipofibroblasts in human lung. *Exp. Lung Res.* 32 379–393. 10.1080/01902140600880257 17090478

[B39] Renier G. Skamene E. DeSanctis J. B. Radzioch D. (1994). Induction of tumor necrosis factor alpha gene expression by lipoprotein lipase. *J. Lipid Res.* 35 271–278. 8169531

[B40] Schmidt B. A. Horsley V. (2013). Intradermal adipocytes mediate fibroblast recruitment during skin wound healing. *Development* 140 1517–1527. 10.1242/dev.087593 23482487 PMC3596993

[B41] Seo M. J. Choi H. S. Jeon H. J. Woo M. S. Lee B. Y. (2014). Baicalein inhibits lipid accumulation by regulating early adipogenesis and m-TOR signaling. *Food Chem. Toxicol.* 67 57–64. 10.1016/j.fct.2014.02.009 24560969

[B42] Shindo S. Hosokawa Y. Hosokawa I. Ozaki K. Matsuo T. (2014). Genipin inhibits MMP-1 and MMP-3 release from TNF-a-stimulated human periodontal ligament cells. *Biochimie* 107(Pt B) 391–395. 10.1016/j.biochi.2014.10.008 25457105

[B43] Takeda Y. Harada Y. Yoshikawa T. Dai P. (2017). Direct conversion of human fibroblasts to brown adipocytes by small chemical compounds. *Sci. Rep.* 7:4304. 10.1038/s41598-017-04665-x 28655922 PMC5487346

[B44] Tsai M. J. Wu P. C. Huang Y. B. Chang J. S. Lin C. L. Tsai Y. H. (2012). Baicalein loaded in tocol nanostructured lipid carriers (tocol NLCs) for enhanced stability and brain targeting. *Int. J. Pharm.* 423 461–470. 10.1016/j.ijpharm.2011.12.009 22193056

[B45] Wang P. Mariman E. Keijer J. Bouwman F. Noben J. P. Robben J. (2004). Profiling of the secreted proteins during 3T3-L1 adipocyte differentiation leads to the identification of novel adipokines. *Cell Mol. Life Sci.* 61 2405–2417. 10.1007/s00018-004-4256-z 15378209 PMC11138943

[B46] Wojciechowicz K. Gledhill K. Ambler C. A. Manning C. B. Jahoda C. A. (2013). Development of the mouse dermal adipose layer occurs independently of subcutaneous adipose tissue and is marked by restricted early expression of FABP4. *PLoS One* 8:e59811. 10.1371/journal.pone.0059811 23555789 PMC3608551

[B47] Xu H. Hertzel A. V. Steen K. A. Wang Q. Suttles J. Bernlohr D. A. (2015). Uncoupling lipid metabolism from inflammation through fatty acid binding protein-dependent expression of UCP2. *Mol. Cell. Biol.* 35 1055–1065. 10.1128/MCB.01122-14 25582199 PMC4333098

[B48] Yeh C. C. Su Y. H. Lin Y. J. Chen P. J. Shi C. S. Chen C. N. (2015). Evaluation of the protective effects of curcuminoid (curcumin and bisdemethoxycurcumin)-loaded liposomes against bone turnover in a cell-based model of osteoarthritis. *Drug Des. Devel. Ther.* 9 2285–2300. 10.2147/DDDT.S78277 25945040 PMC4408943

[B49] Zeng C. Jiang W. Tan M. Yang X. He C. Huang W. (2016). Optimization of the process variables of tilianin-loaded composite phospholipid liposomes based on response surface-central composite design and pharmacokinetic study. *Eur. J. Pharm. Sci.* 85 123–131. 10.1016/j.ejps.2016.02.007 26883760

