# Supplementary figures and images for: Liposome-Encapsulated Baicalein Suppressed Lipogenesis and Extracellular Matrix Formation in Hs68 Human Dermal Fibroblasts
Source: Front Pharmacol. 2018 Mar 6;9:155. doi: 10.3389/fphar.2018.00155 (PMC5845745; doi:10.3389/fphar.2018.00155)

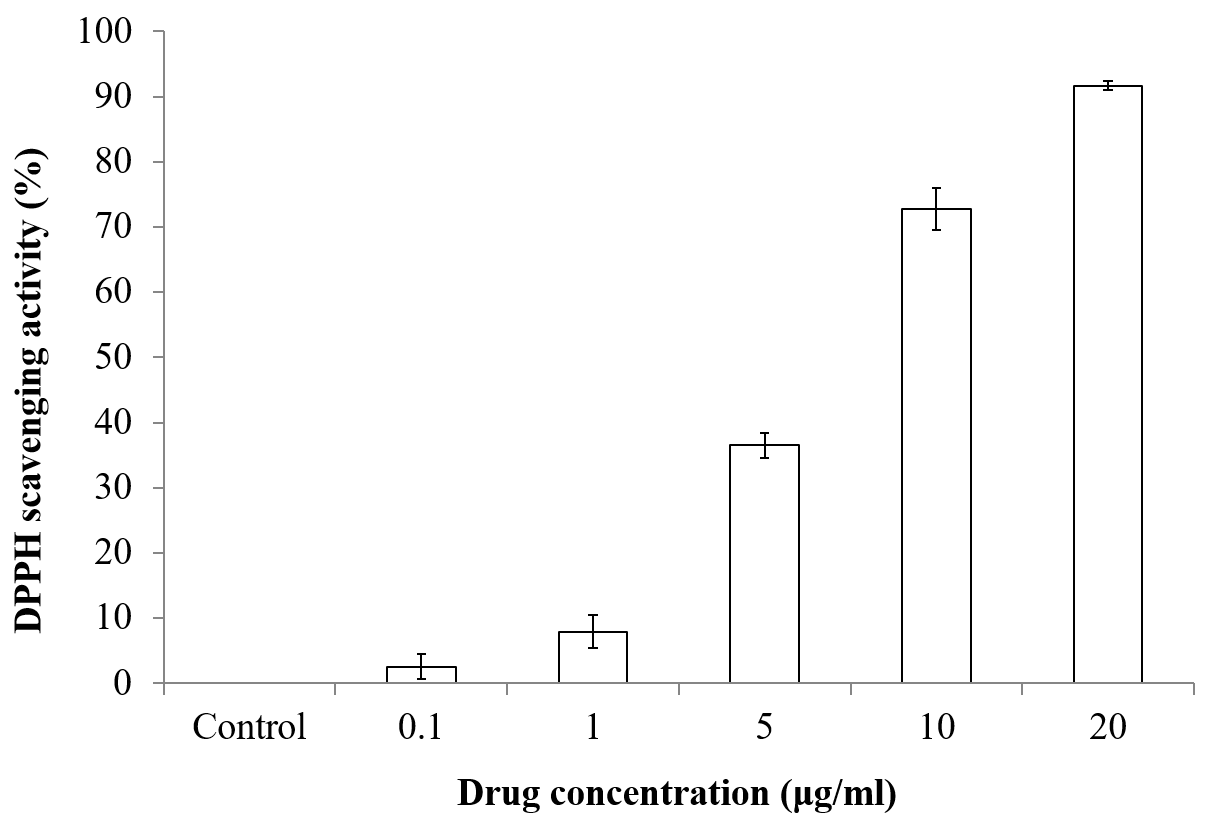

Supplement: FIGURE S1 — The anti-oxidant activity of baicalein measured by DPPH scavenging assay. [file Image_1.TIF]
